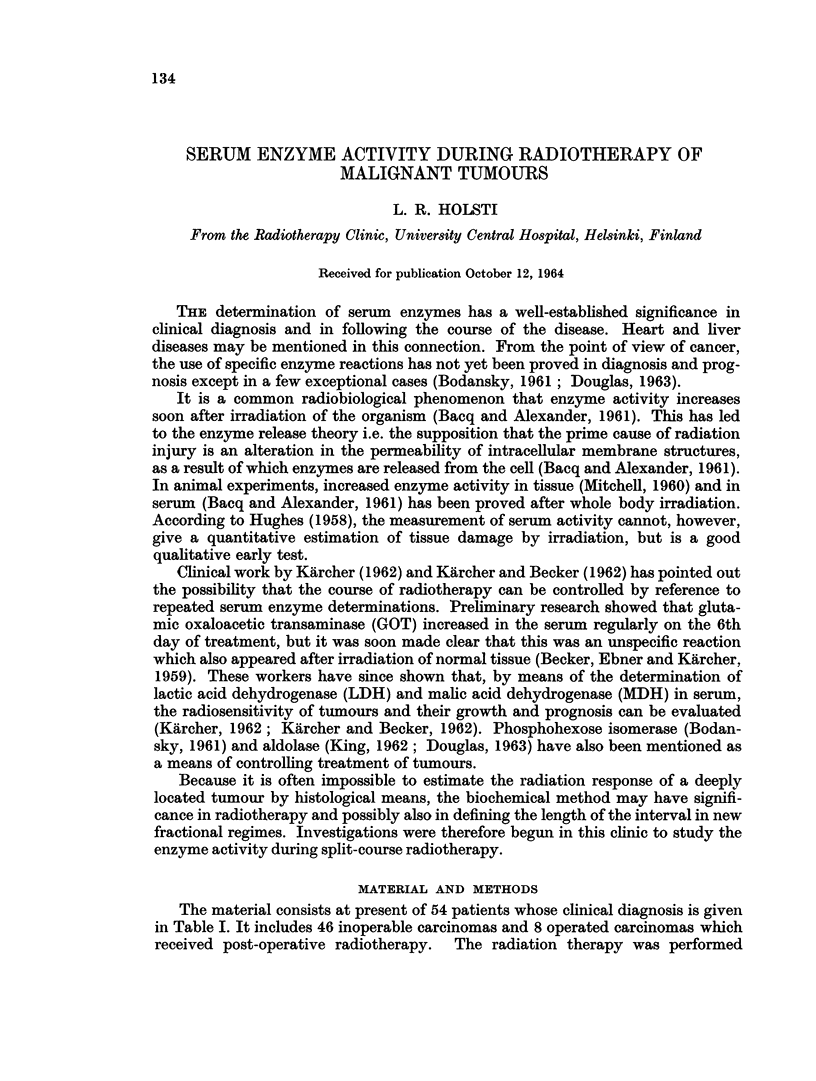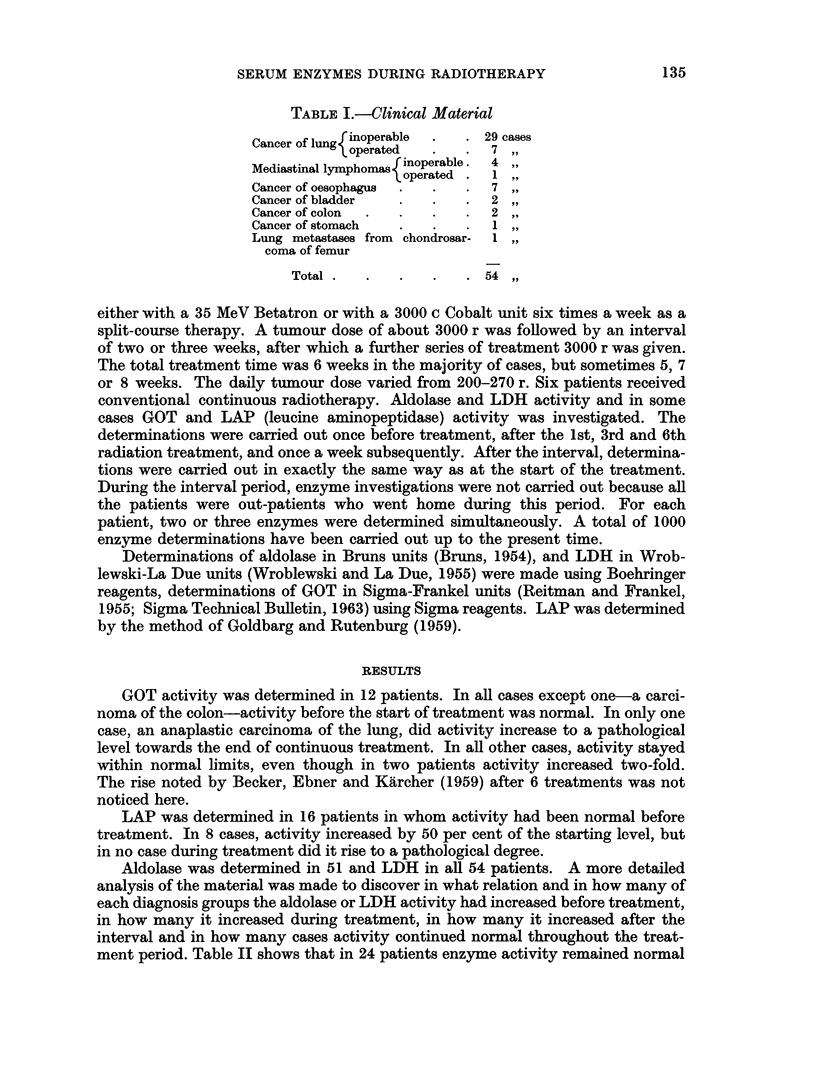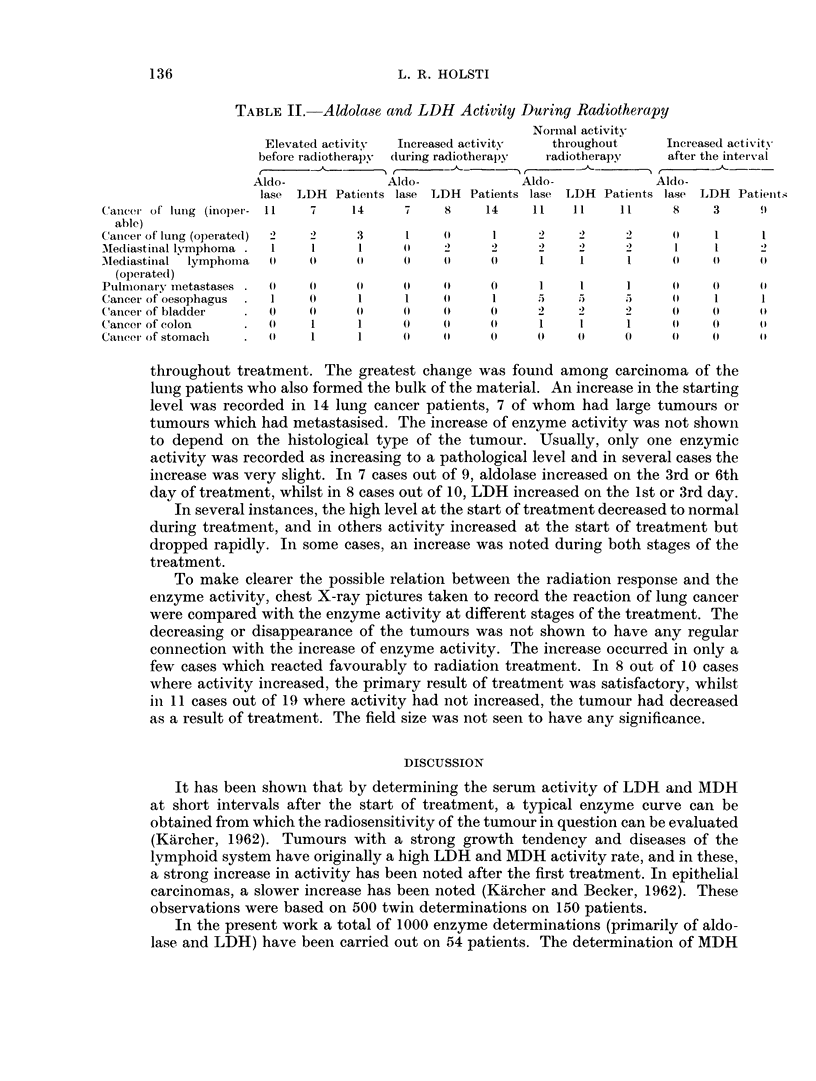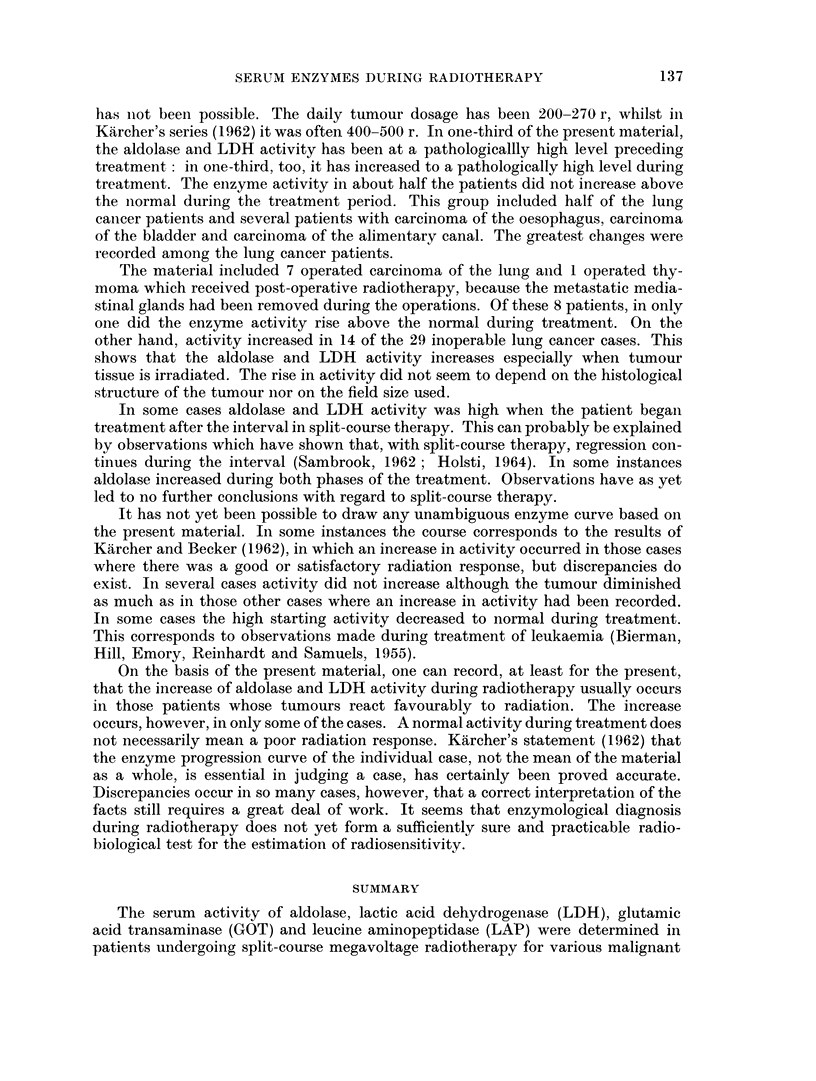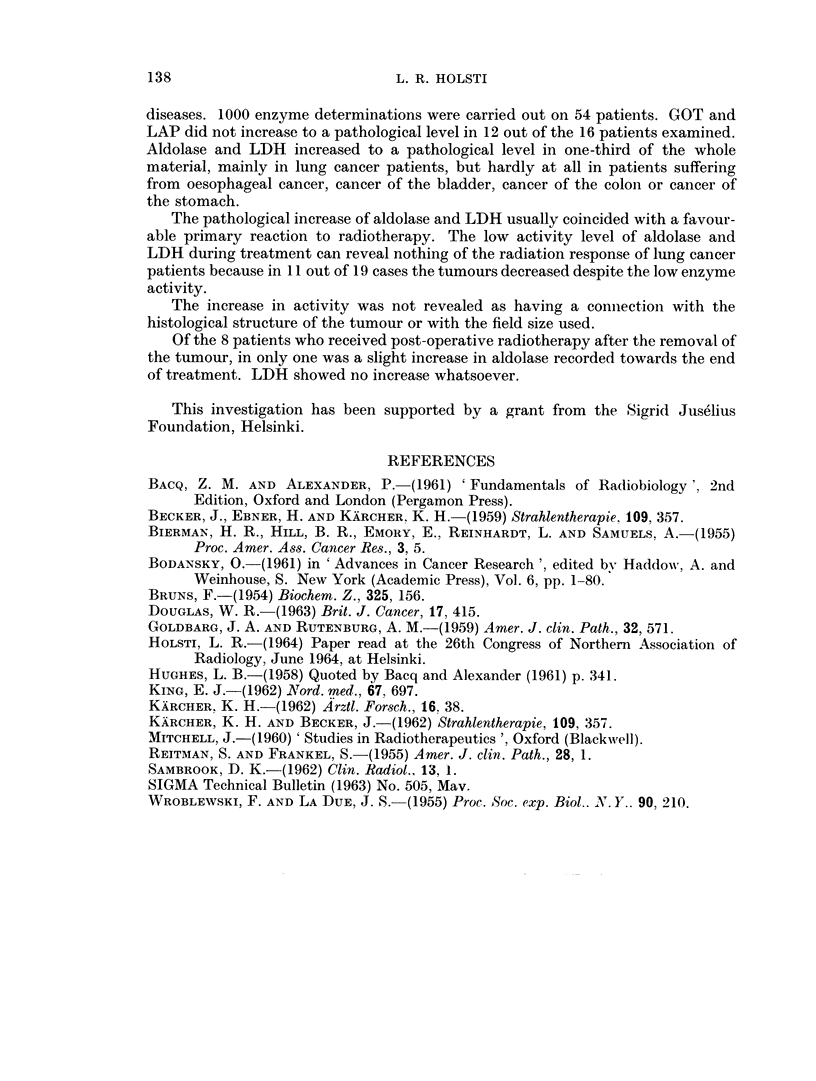# Serum Enzyme Activity During Radiotherapy of Malignant Tumours

**DOI:** 10.1038/bjc.1965.15

**Published:** 1965-03

**Authors:** L. R. Holsti


### SERUM ENZYME ACTIVITY DURING RADIOTHERAPY OF MALIGNANT TUMOURS

L. R. HOLSTI

*From the Radiotherapy Clinic, University Central Hospital, Helsinki, Finland*

Received for publication October 12, 1964

THE determination of serum enzymes has a well-established significance in clinical diagnosis and in following the course of the disease. Heart and liver diseases may be mentioned in this connection. From the point of view of cancer, the use of specific enzyme reactions has not yet been proved in diagnosis and prognosis except in a few exceptional cases (Bodansky, 1961 ; Douglas, 1963).

It is a common radiobiological phenomenon that enzyme activity increases soon after irradiation of the organism (Bacq and Alexander, 1961). This has led to the enzyme release theory i.e. the supposition that the prime cause of radiation injury is an alteration in the permeability of intracellular membrane structures, as a result of which enzymes are released from the cell (Bacq and Alexander, 1961). In animal experiments, increased enzyme activity in tissue (Mitchell, 1960) and in serum (Bacq and Alexander, 1961) has been proved after whole body irradiation. According to Hughes (1958), the measurement of serum activity cannot, however, give a quantitative estimation of tissue damage by irradiation, but is a good qualitative early test.

Clinical work by Kärcher (1962) and Kärcher and Becker (1962) has pointed out the possibility that the course of radiotherapy can be controlled by reference to repeated serum enzyme determinations. Preliminary research showed that glutamic oxaloacetic transaminase (GOT) increased in the serum regularly on the 6th day of treatment, but it was soon made clear that this was an unspecific reaction which also appeared after irradiation of normal tissue (Becker, Ebner and Kärcher, 1959). These workers have since shown that, by means of the determination of lactic acid dehydrogenase (LDH) and malic acid dehydrogenase (MDH) in serum, the radiosensitivity of tumours and their growth and prognosis can be evaluated (Kärcher, 1962 ; Kärcher and Becker, 1962). Phosphohexose isomerase (Bodansky, 1961) and aldolase (King, 1962 ; Douglas, 1963) have also been mentioned as a means of controlling treatment of tumours.

Because it is often impossible to estimate the radiation response of a deeply located tumour by histological means, the biochemical method may have significance in radiotherapy and possibly also in defining the length of the interval in new fractional regimes. Investigations were therefore begun in this clinic to study the enzyme activity during split-course radiotherapy.

### MATERIAL AND METHODS

The material consists at present of 54 patients whose clinical diagnosis is given in Table I. It includes 46 inoperable carcinomas and 8 operated carcinomas which received post-operative radiotherapy. The radiation therapy was performed

TABLE I.—*Clinical Material*

| | | |
|---|---|---|
| Cancer of lung | inoperable | 29 cases |
| | operated | 7 ,, |
| Mediastinal lymphomas | inoperable | 4 ,, |
| | operated | 1 ,, |
| Cancer of oesophagus | | 7 ,, |
| Cancer of bladder | | 2 ,, |
| Cancer of colon | | 2 ,, |
| Cancer of stomach | | 1 ,, |
| Lung metastases from chondrosarcoma of femur | | 1 ,, |
| Total | | 54 ,, |

either with a 35 MeV Betatron or with a 3000 c Cobalt unit six times a week as a split-course therapy. A tumour dose of about 3000 r was followed by an interval of two or three weeks, after which a further series of treatment 3000 r was given. The total treatment time was 6 weeks in the majority of cases, but sometimes 5, 7 or 8 weeks. The daily tumour dose varied from 200–270 r. Six patients received conventional continuous radiotherapy. Aldolase and LDH activity and in some cases GOT and LAP (leucine aminopeptidase) activity was investigated. The determinations were carried out once before treatment, after the 1st, 3rd and 6th radiation treatment, and once a week subsequently. After the interval, determinations were carried out in exactly the same way as at the start of the treatment. During the interval period, enzyme investigations were not carried out because all the patients were out-patients who went home during this period. For each patient, two or three enzymes were determined simultaneously. A total of 1000 enzyme determinations have been carried out up to the present time.

Determinations of aldolase in Bruns units (Bruns, 1954), and LDH in Wroblewski-La Due units (Wroblewski and La Due, 1955) were made using Boehringer reagents, determinations of GOT in Sigma-Frankel units (Reitman and Frankel, 1955; Sigma Technical Bulletin, 1963) using Sigma reagents. LAP was determined by the method of Goldbarg and Rutenburg (1959).

### RESULTS

GOT activity was determined in 12 patients. In all cases except one—a carcinoma of the colon—activity before the start of treatment was normal. In only one case, an anaplastic carcinoma of the lung, did activity increase to a pathological level towards the end of continuous treatment. In all other cases, activity stayed within normal limits, even though in two patients activity increased two-fold. The rise noted by Becker, Ebner and Kärcher (1959) after 6 treatments was not noticed here.

LAP was determined in 16 patients in whom activity had been normal before treatment. In 8 cases, activity increased by 50 per cent of the starting level, but in no case during treatment did it rise to a pathological degree.

Aldolase was determined in 51 and LDH in all 54 patients. A more detailed analysis of the material was made to discover in what relation and in how many of each diagnosis groups the aldolase or LDH activity had increased before treatment, in how many it increased during treatment, in how many it increased after the interval and in how many cases activity continued normal throughout the treatment period. Table II shows that in 24 patients enzyme activity remained normal

TABLE II.—*Aldolase and LDH Activity During Radiotherapy*

| | Elevated activity before radiotherapy | | | Increased activity during radiotherapy | | | Normal activity throughout radiotherapy | | | Increased activity after the interval | | |
|---|---|---|---|---|---|---|---|---|---|---|---|---|
| | Aldolase | LDH | Patients | Aldolase | LDH | Patients | Aldolase | LDH | Patients | Aldolase | LDH | Patients |
| Cancer of lung (inoperable) | 11 | 7 | 14 | 7 | 8 | 14 | 11 | 11 | 11 | 8 | 3 | 9 |
| Cancer of lung (operated) | 2 | 2 | 3 | 1 | 0 | 1 | 2 | 2 | 2 | 0 | 1 | 1 |
| Mediastinal lymphoma . | 1 | 1 | 1 | 0 | 2 | 2 | 2 | 2 | 2 | 1 | 1 | 2 |
| Mediastinal lymphoma (operated) | 0 | 0 | 0 | 0 | 0 | 0 | 1 | 1 | 1 | 0 | 0 | 0 |
| Pulmonary metastases . | 0 | 0 | 0 | 0 | 0 | 0 | 1 | 1 | 1 | 0 | 0 | 0 |
| Cancer of oesophagus . | 1 | 0 | 1 | 1 | 0 | 1 | 5 | 5 | 5 | 0 | 1 | 1 |
| Cancer of bladder . | 0 | 0 | 0 | 0 | 0 | 0 | 2 | 2 | 2 | 0 | 0 | 0 |
| Cancer of colon . | 0 | 1 | 1 | 0 | 0 | 0 | 1 | 1 | 1 | 0 | 0 | 0 |
| Cancer of stomach . | 0 | 1 | 1 | 0 | 0 | 0 | 0 | 0 | 0 | 0 | 0 | 0 |

throughout treatment. The greatest change was found among carcinoma of the lung patients who also formed the bulk of the material. An increase in the starting level was recorded in 14 lung cancer patients, 7 of whom had large tumours or tumours which had metastasised. The increase of enzyme activity was not shown to depend on the histological type of the tumour. Usually, only one enzymic activity was recorded as increasing to a pathological level and in several cases the increase was very slight. In 7 cases out of 9, aldolase increased on the 3rd or 6th day of treatment, whilst in 8 cases out of 10, LDH increased on the 1st or 3rd day.

In several instances, the high level at the start of treatment decreased to normal during treatment, and in others activity increased at the start of treatment but dropped rapidly. In some cases, an increase was noted during both stages of the treatment.

To make clearer the possible relation between the radiation response and the enzyme activity, chest X-ray pictures taken to record the reaction of lung cancer were compared with the enzyme activity at different stages of the treatment. The decreasing or disappearance of the tumours was not shown to have any regular connection with the increase of enzyme activity. The increase occurred in only a few cases which reacted favourably to radiation treatment. In 8 out of 10 cases where activity increased, the primary result of treatment was satisfactory, whilst in 11 cases out of 19 where activity had not increased, the tumour had decreased as a result of treatment. The field size was not seen to have any significance.

### DISCUSSION

It has been shown that by determining the serum activity of LDH and MDH at short intervals after the start of treatment, a typical enzyme curve can be obtained from which the radiosensitivity of the tumour in question can be evaluated (Kärcher, 1962). Tumours with a strong growth tendency and diseases of the lymphoid system have originally a high LDH and MDH activity rate, and in these, a strong increase in activity has been noted after the first treatment. In epithelial carcinomas, a slower increase has been noted (Kärcher and Becker, 1962). These observations were based on 500 twin determinations on 150 patients.

In the present work a total of 1000 enzyme determinations (primarily of aldolase and LDH) have been carried out on 54 patients. The determination of MDH

has not been possible. The daily tumour dosage has been 200–270 r, whilst in Kärcher's series (1962) it was often 400–500 r. In one-third of the present material, the aldolase and LDH activity has been at a pathologicallly high level preceding treatment : in one-third, too, it has increased to a pathologically high level during treatment. The enzyme activity in about half the patients did not increase above the normal during the treatment period. This group included half of the lung cancer patients and several patients with carcinoma of the oesophagus, carcinoma of the bladder and carcinoma of the alimentary canal. The greatest changes were recorded among the lung cancer patients.

The material included 7 operated carcinoma of the lung and 1 operated thymoma which received post-operative radiotherapy, because the metastatic mediastinal glands had been removed during the operations. Of these 8 patients, in only one did the enzyme activity rise above the normal during treatment. On the other hand, activity increased in 14 of the 29 inoperable lung cancer cases. This shows that the aldolase and LDH activity increases especially when tumour tissue is irradiated. The rise in activity did not seem to depend on the histological structure of the tumour nor on the field size used.

In some cases aldolase and LDH activity was high when the patient began treatment after the interval in split-course therapy. This can probably be explained by observations which have shown that, with split-course therapy, regression continues during the interval (Sambrook, 1962 ; Holsti, 1964). In some instances aldolase increased during both phases of the treatment. Observations have as yet led to no further conclusions with regard to split-course therapy.

It has not yet been possible to draw any unambiguous enzyme curve based on the present material. In some instances the course corresponds to the results of Kärcher and Becker (1962), in which an increase in activity occurred in those cases where there was a good or satisfactory radiation response, but discrepancies do exist. In several cases activity did not increase although the tumour diminished as much as in those other cases where an increase in activity had been recorded. In some cases the high starting activity decreased to normal during treatment. This corresponds to observations made during treatment of leukaemia (Bierman, Hill, Emory, Reinhardt and Samuels, 1955).

On the basis of the present material, one can record, at least for the present, that the increase of aldolase and LDH activity during radiotherapy usually occurs in those patients whose tumours react favourably to radiation. The increase occurs, however, in only some of the cases. A normal activity during treatment does not necessarily mean a poor radiation response. Kärcher's statement (1962) that the enzyme progression curve of the individual case, not the mean of the material as a whole, is essential in judging a case, has certainly been proved accurate. Discrepancies occur in so many cases, however, that a correct interpretation of the facts still requires a great deal of work. It seems that enzymological diagnosis during radiotherapy does not yet form a sufficiently sure and practicable radiobiological test for the estimation of radiosensitivity.

### SUMMARY

The serum activity of aldolase, lactic acid dehydrogenase (LDH), glutamic acid transaminase (GOT) and leucine aminopeptidase (LAP) were determined in patients undergoing split-course megavoltage radiotherapy for various malignant diseases. 1000 enzyme determinations were carried out on 54 patients. GOT and LAP did not increase to a pathological level in 12 out of the 16 patients examined. Aldolase and LDH increased to a pathological level in one-third of the whole material, mainly in lung cancer patients, but hardly at all in patients suffering from oesophageal cancer, cancer of the bladder, cancer of the colon or cancer of the stomach.

The pathological increase of aldolase and LDH usually coincided with a favourable primary reaction to radiotherapy. The low activity level of aldolase and LDH during treatment can reveal nothing of the radiation response of lung cancer patients because in 11 out of 19 cases the tumours decreased despite the low enzyme activity.

The increase in activity was not revealed as having a connection with the histological structure of the tumour or with the field size used.

Of the 8 patients who received post-operative radiotherapy after the removal of the tumour, in only one was a slight increase in aldolase recorded towards the end of treatment. LDH showed no increase whatsoever.

This investigation has been supported by a grant from the Sigrid Jusélius Foundation, Helsinki.

### REFERENCES

Bacq, Z. M. and Alexander, P.—(1961) ‘ Fundamentals of Radiobiology ’, 2nd Edition, Oxford and London (Pergamon Press).
Becker, J., Ebner, H. and Kärcher, K. H.—(1959) *Strahlentherapie*, **109**, 357.
Bierman, H. R., Hill, B. R., Emory, E., Reinhardt, L. and Samuels, A.—(1955) *Proc. Amer. Ass. Cancer Res.*, **3**, 5.
Bodansky, O.—(1961) in ‘ Advances in Cancer Research ’, edited by Haddow, A. and Weinhouse, S. New York (Academic Press), Vol. 6, pp. 1–80.
Bruns, F.—(1954) *Biochem. Z.*, **325**, 156.
Douglas, W. R.—(1963) *Brit. J. Cancer*, **17**, 415.
Goldbarg, J. A. and Rutenburg, A. M.—(1959) *Amer. J. clin. Path.*, **32**, 571.
Holsti, L. R.—(1964) Paper read at the 26th Congress of Northern Association of Radiology, June 1964, at Helsinki.
Hughes, L. B.—(1958) Quoted by Bacq and Alexander (1961) p. 341.
King, E. J.—(1962) *Nord. med.*, **67**, 697.
Kärcher, K. H.—(1962) *Ärztl. Forsch.*, **16**, 38.
Kärcher, K. H. and Becker, J.—(1962) *Strahlentherapie*, **109**, 357.
Mitchell, J.—(1960) ‘ Studies in Radiotherapeutics ’, Oxford (Blackwell).
Reitman, S. and Frankel, S.—(1955) *Amer. J. clin. Path.*, **28**, 1.
Sambrook, D. K.—(1962) *Clin. Radiol.*, **13**, 1.
SIGMA Technical Bulletin (1963) No. 505, May.
Wroblewski, F. and La Due, J. S.—(1955) *Proc. Soc. exp. Biol., N.Y.*, **90**, 210.